# Artificial intelligence–based quantification of epidermal proliferation and apoptosis in human skin

**DOI:** 10.1016/j.xjidi.2026.100509

**Published:** 2026-07-16

**Authors:** Valeria Mei, Sandra Maurer, Philipp Kainz, Andreas Ettner-Sitter, Thea Bauer, Thiha Aung, Silke Haerteis, Vera Rötzer

**Affiliations:** 1 Institute for Molecular and Cellular Anatomy, University of Regensburg, Regensburg, Germany; 2 KOLAIDO GmbH, Altenrhein, Switzerland; 3 Faculty of Applied Healthcare Science, Deggendorf Institute of Technology, Deggendorf, Germany

**Keywords:** CASP3, Convolutional neuronal networks, Epidermis, Hydroxytyrosol, Ki-67

## Abstract

Although artificial intelligence is rapidly advancing, its application in translational dermatological research remains limited. In this study, we established an artificial intelligence–based image analysis workflow for the quantification of proliferation and apoptosis within the human epidermis using 3,3′-diaminobenzidine–based immunohistochemical staining. Human skin cultured in a 3-dimensional in vivo model was processed using paraffin embedding and immunohistochemical staining for Ki-67 and cleaved Caspase-3. We implemented an artificial intelligence–based 2-model workflow: a custom-trained convolutional neural network–based model for cell detection and a semantic-segmentation model that specifically segregates the epidermis. By combining both models, we restricted cell annotation and classification to the epidermis—our structure of interest—enabling epidermis-constrained quantification on whole-slide images. Validation against manual counts showed high agreement for both markers (Ki-67 mean accuracy = 95.43%; cleaved Caspase-3 = 97.0%) across staining batches and time points. To test the experimental applicability of our workflow, we treated human skin cultured on the chorioallantoic membrane with hydroxytyrosol. The artificial intelligence–based workflow minimized subjective bias and provided a coherent readout, showing reduced proliferation without inducing apoptotic responses in hydroxytyrosol-treated samples. Overall, the workflow achieved strong performance with modest training effort and annotation, offering a scalable approach that supports translational dermatological research and holds potential for dermatopathological applications.

## Introduction

Histological image analysis is a crucial part of pathology, enabling insights into tissue morphology, cellular organization, and molecular markers that guide research and clinical diagnostics. Traditionally, assessment of histological specimens relies on expert visual inspection under a microscope, which is a time-consuming process susceptible to subjective interpretation (Doğan and Yılmaz, 2023). This poses a substantial challenge, especially as the scale and complexity of tissue-based studies continue to increase. With the advent of digital pathology and whole-slide images (WSIs), high-resolution digitalization of tissue sections has become feasible, enabling systematic visualization and computational analysis of histological datasets (Pantanowitz, 2010). Still, the volume and heterogeneity of histological data introduce further challenges that require robust computational methods to improve data processing.

The integration of artificial intelligence (AI) has already started to increase the accuracy and efficiency of diagnostic workflows. For instance, the diagnostic accuracy of AI-based image analysis has been demonstrated for cancer classifications of tumors in the gastrointestinal tract and for assessing breast cancer (Dy et al, 2024; Iizuka et al, 2020; Park et al, 2021).

The most suitable AI models for histological image analysis are predominantly deep learning–based neural network architectures, in particular, convolutional neural networks (CNNs). CNNs are highly suitable for processing image input data, because they are able to recognize and classify different visual features such as cellular structures and tissue types (Bejnordi et al, 2017). The working principle behind CNNs is based on findings from the hierarchical organization of the cat visual cortex, because they consist of multiple layers and convolutions, similar to neurons (Hubel and Wiesel, 1959). These layers are modeled as a mathematical convolution operation and respond like filters to the most informative patterns, such as shapes and colors. These are then further processed as more complex representations in deeper layers of the network to identify specific objects or inform a classification of an image (Dabeer et al, 2019; Sarvamangala and Kulkarni, 2022). Although a broader variety of AI models exists, CNNs are presumably the preferred technique for computer vision and image analysis applications (Litjens et al, 2017).

In the field of histology and histopathology, AI-based applications are being introduced to autonomously interpret histological data from cancer tissue, assisting manual analysis and offering consistent results (Wang et al, 2025). By contrast, the application of AI in dermatopathology and skin histology remains more limited (Vidal et al, 2023).

Applications of AI-based methods in the field of dermatology require a clear understanding of skin morphology, with skin being the largest organ of the human body and serving as a crucial primary barrier to the environment. It can be divided into the dermal layer, which is responsible for the skin’s mechanical properties and consists mostly of fibroblasts and collagen fibers, and the outer layer, called the epidermis. The latter is a rather dense network primarily consisting of keratinocytes that mature over time and ascend to the surface (Menon, 2002).

Skin pathologies, such as chronic wounds, can have far-reaching effects on patients and place a substantial burden on society and healthcare systems (Gu et al, 2021; Guest et al, 2017; Sen, 2025; Sen et al, 2009). At the tissue level, many dermatological conditions are characterized by dysregulated epidermal homeostasis, which is reflected by altered rates of keratinocyte proliferation and apoptosis (Bebars et al, 2017; Ohno et al, 2021).

Although notable progress in the implementation of AI-based methods has been made, with a primary focus on the classification of skin cancers, AI-assisted image analysis holds considerable potential beyond oncologic applications (Blum et al, 2004; Li et al, 2022b).

Extending AI-based analytical approaches could therefore provide substantial benefits. Indeed, AI is being introduced to support clinical diagnosis and disease characterization, particularly in inflammatory skin diseases such as atopic dermatitis, psoriasis, acne, and rheumatic skin conditions (Ding et al, 2025; Goessinger et al, 2024; Huang et al, 2023; Tang et al, 2026). In addition, AI-based approaches have been proposed for treatment response assessment and wound care applications (Ganesan et al, 2024; Schwarz, 2025).

Although these advances largely focus on clinical image analysis, histopathological examination remains the diagnostic gold standard and could also benefit from AI-based methods (Sopjani et al, 2022). Existing work has shown promising results for specific histological applications, including wound healing and psoriasis, but the application of these approaches across dermatological indications is still at an early stage (Della Mura et al, 2025; He et al, 2025; Jones and Quinn, 2021; Kerkour et al, 2025).

This highlights the need for further research into AI-based methods for dermatological histology and the requirement for suitable experimental models for human skin to enable such research. In this context, alternative models such as the chorioallantoic membrane (CAM) model are receiving increasing attention as a promising platform to perform in vivo analyses of human skin (Maurer et al, unpublished data). Furthermore, the CAM model incorporates the 3R principles (“Replace, Reduce, Refine”), because it offers an attractive alternative to animal models and helps to reduce them (Nowak-Sliwinska et al, 2014).

A particular interest in skin biology is the balance between proliferation and apoptosis, which is fundamental for maintaining tissue homeostasis and enabling dynamic adaptation, as the skin undergoes continuous regeneration and adapts to environmental stressors (Li et al, 2022a). Two molecular markers are frequently used for the investigation of these opposing cellular processes: Ki-67, a well-established proliferation marker, and cleaved Caspase-3 (CASP3), an important player in the apoptotic cascade (Durgun et al, 2023).

Ki-67 is a nuclear antigen expressed in all phases of the cell cycle (G1, S, G2, and M phase) except the quiescent state (G0) and peaks during mitosis (Lopez et al, 1991; Scholzen and Gerdes, 2000). In the epidermis of the skin, most Ki-67–positive cells are found in the basal layers and indicate ongoing regeneration processes (Petrovic et al, 2018; Rakita et al, 2020). Cleaved CASP3, on the other hand, is a member of the endoproteases and is cleaved to transition into its active form within cells undergoing programmed cell death. Immunohistochemical (IHC) detection of activated cleaved CASP3 is widely used to identify apoptotic cells in skin sections and provides insights into physiological processes such as epidermal renewal and pathological states such as inflammation and injury. Typically, cleaved CASP3 expression in healthy skin is rather low but increases in response to stressors such as UVR and mechanical injury (Ahmadi Ashtiani et al, 2019; Del Duca et al, 2025).

A known modulator of proliferation and apoptosis is the phenolic compound hydroxytyrosol (HT), which is found in olive oil (El-Azem et al, 2019; Notarnicola et al, 2011). Although its inhibitory effect on proliferation in cancer cells has been shown, studies have also discussed its proproliferative properties, indicating potential utility for wound healing (Granados-Principal et al, 2011; Zrelli et al, 2011).

In this study, we established an AI-based image analysis workflow using the IKOSA platform (KOLAIDO GmbH, Altenrhein, Switzerland) as a valuable addition to our wound healing research. This image analysis pipeline enables accurate quantification of IHC markers such as Ki-67 and cleaved CASP3 to gain deeper insights into human skin regeneration and the evaluation of potential modulators such as HT in the epidermal skin tissue.

## Results

**Figure 1:**
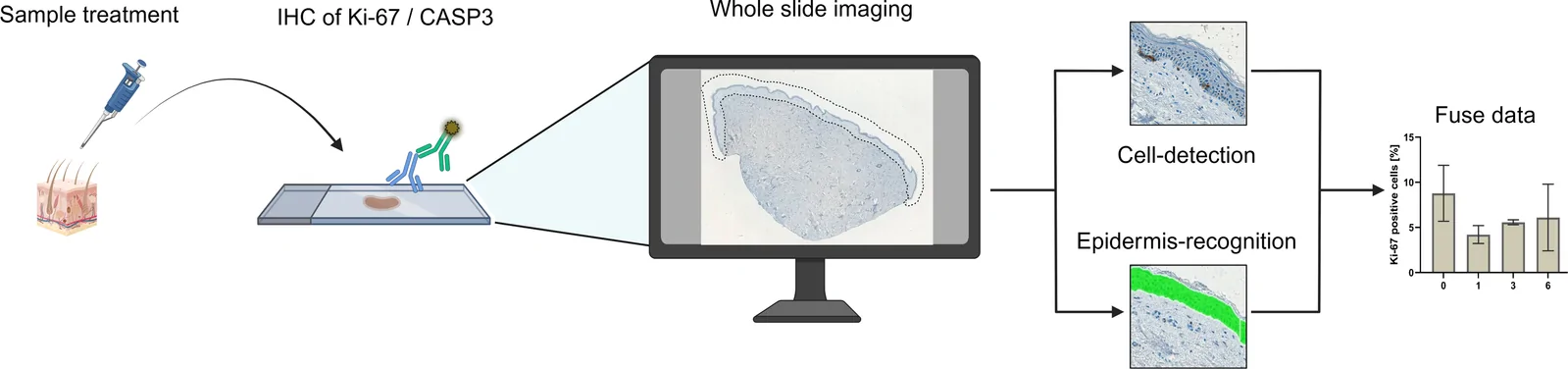
**Overview of the workflow.** Human skin samples were treated during cultivation and IHC stained after excision. Slides were digitized and analyzed with 2 combined AI-based models annotating cells and the epidermis. Image was created in BioRender (Maurer, 2026; https://BioRender.com/l4zcn4j). AI, artificial intelligence; IHC, immunohistochemically.

In this study, we employed AI-based algorithms to automatically quantify proliferating and apoptotic cells in the epidermis of human skin after cultivation on the CAM. The analysis pipeline was developed for IHC-stained WSIs by combining an AI model for cell detection and another AI model that recognizes the nucleated part of the epidermis, our structure of interest. This optimized pipeline was then applied to assess the impact of topical HT treatment on human skin, as outlined in Figure 1.

### AI cell-detection model

**Figure 2:**
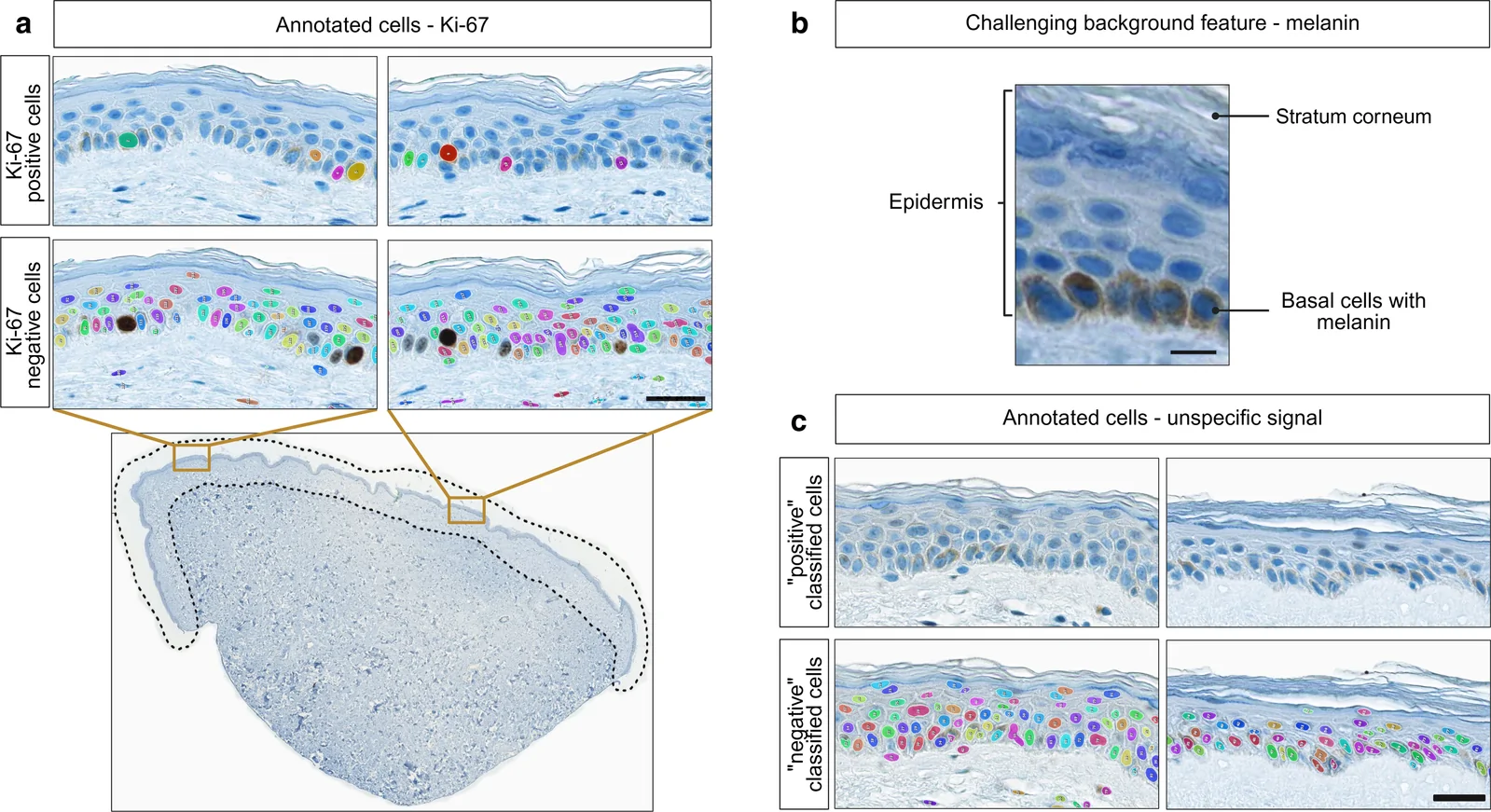
**Ki-67–positive and –negative cells annotated by the AI model for cell detection (multicolored ellipses).** (**a**) Proliferating epidermal cells were annotated and classified by the AI model. Bar = 25 μm. (**b**) Negative control showing unspecific background features such as melanin, primarily in the basal epidermal layer. Bar = 10 μm. (**c**) Background features were not annotated or misclassified by the AI model. Bar = 50 μm. AI, artificial intelligence.

Visualized prediction overlays showed that the AI cell-detection model produced highly plausible and visually consistent cell annotations. It distinguished between Ki-67–positive and –negative cells, giving an overall strong impression of the model’s performance (Figure 2a).

In addition, in the absence of specific immunostaining, we observed no annotation of background features, such as melanin pigment in the epidermis (Figure 2b and c), indicating the specificity of the AI model.

To complement the qualitative results, quantitative training validation was performed on 16 manually annotated regions of interest (ROIs) covering the biological variation in sections stained for Ki-67. In contrast to the qualitative assessment, the validation metrics revealed an unsatisfactory false-positive rate, and the values for pixel-wise precision and recall did not reach our internally predefined minimum performance target (Table 1).

**Table 1:** Quantification of the Initial Training Validation of the AI Model for Cell Detection

Cell Class	Precision (%)	Recall (%)	AP	FP (%)
Negative	79.33	84.74	0.80	18.09
Positive	38.71	28.57	0.28	31.15

A mismatch between manually annotated regions and the overall scope of the model’s detections resulted in a low validation performance.

Abbreviations: AI, artificial intelligence; AP, average precision; FP, false positive.

Upon closer examination, this reduction in measured performance, despite the qualitatively strong detection, was largely attributable to a mismatch between the manual ground truth and the algorithm’s detection scope: the manual annotations included only epidermal cells within the ROI, whereas the model counted cells across the entire ROI, including the dermis. To address this issue with a targeted approach, we trained another dedicated AI model to segment the epidermis, our structure of interest. By combining both AI models, we aimed to restrict subsequent cell detection and quantification exclusively to epidermal regions.

### AI epidermis-recognition model

After training, the epidermis-recognition model was validated on an independent set of 16 ROIs selected to reflect the diversity of epidermal appearance encountered across samples and staining batches. The epidermis-recognition model achieved consistent performance, with a Dice coefficient of 0.96, a pixel-wise precision of 94.95%, a recall of 96.23%, and a specificity of 98.59%. These segmentation metrics were computed by comparing the predictions of the AI model with those of the corresponding manual ground-truth masks.

**Figure 3:**
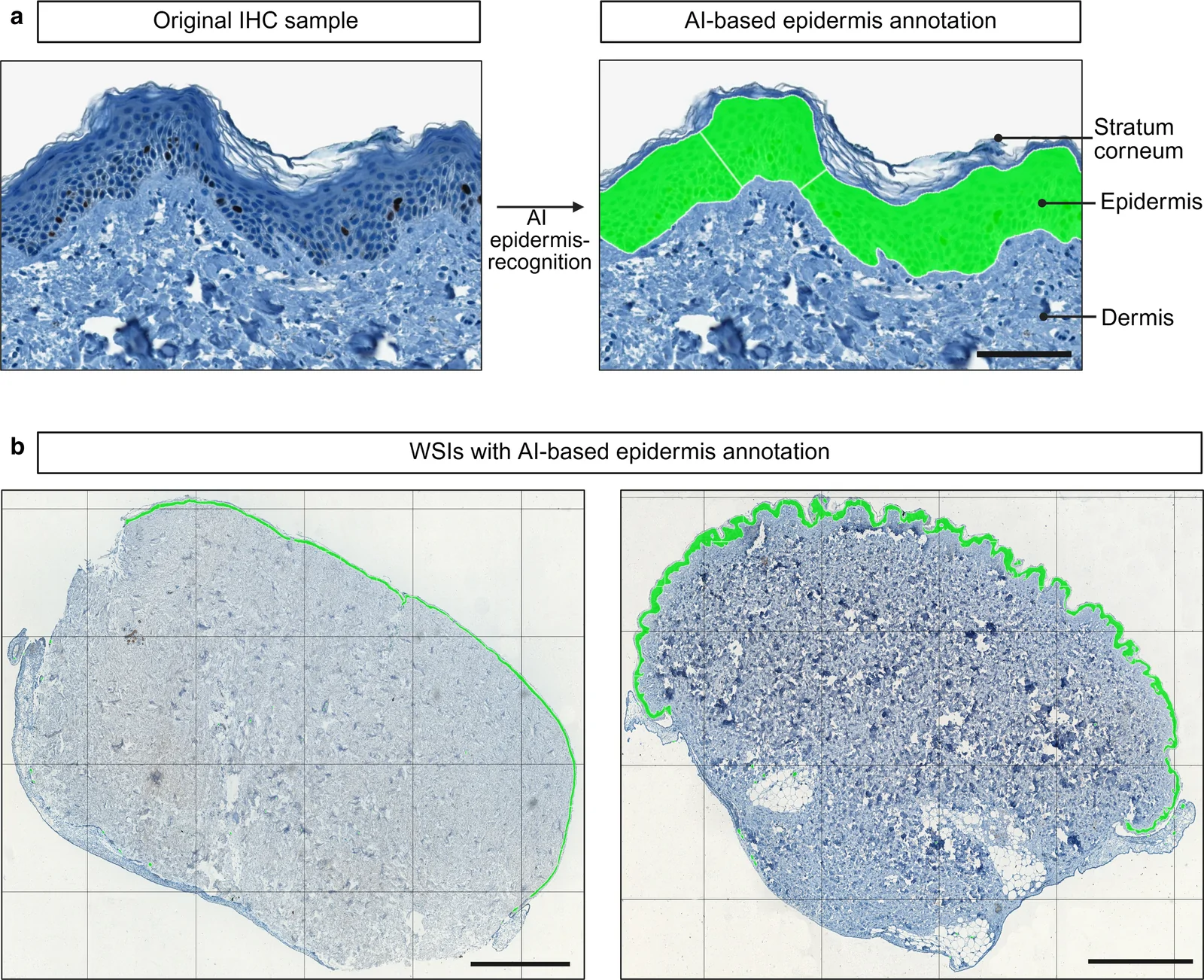
**Epidermis annotated by the AI model (green).** (**a**) The AI model differentiated between dermis and epidermis and annotated the epidermis without including the stratum corneum, as intended. Bar = ∼50 μm. (**b**) At different hematoxylin intensities, the epidermis was correctly annotated in IHC-stained patient tissue. Bar = ∼1 mm. AI, artificial intelligence; IHC, immunohistochemical.

Qualitative evaluation of the prediction overlays from the AI epidermis-recognition model further demonstrated accurate epidermal delineation across samples (Figure 3a). Importantly, experimentally inevitable variability, such as differences in hematoxylin intensity, did not impair the algorithm’s ability to correctly identify the epidermal compartment (Figure 3b). This accurate delineation formed the basis for subsequently restricting cell quantification to epidermal regions only.

### Combined workflow: AI-based epidermis-constrained quantification

#### Manual validation

**Figure 4:**
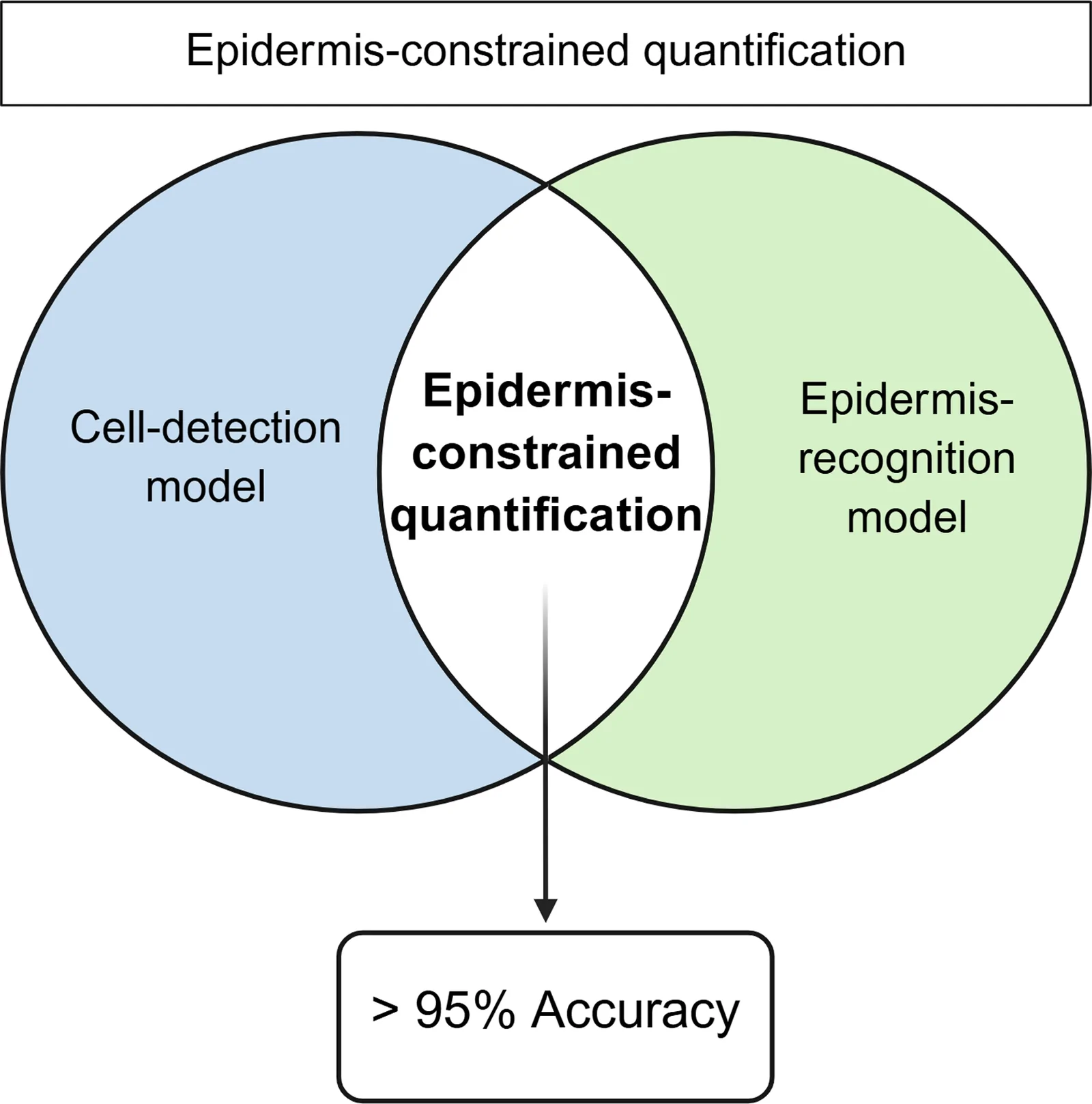
**Schematic visualization of the epidermis-constrained quantification.** By combining the outputs of the AI model for cell detection with the outputs of the model for epidermis recognition, a mean accuracy >95% was achieved. Image was created in BioRender (Maurer, 2026; https://BioRender.com/l4zcn4j). AI, artificial intelligence.

By combining the AI model for cell detection with the other one for epidermis recognition—referred to as epidermis-constrained quantification in the remaining parts of this paper—the specificity and overall quality of downstream measurements were substantially improved (Figure 4).

To validate this combined workflow, we evaluated agreement at the level of positive cell ratios rather than using per-cell confusion matrices. This was based on our goal of assessing overall model performance and the fact that the model was learned to classify positive and negative labels separately, in accordance with studies from Smits et al (2023).

To estimate level misclassification, we approximated the false-positive rate using the relative delta Δ_rel,i_. The Δ_rel,i_ reflects the degree to which the AI model assigns positive labels beyond those identified manually. Its negative values (Table 2) obtained across the validation set demonstrated that, in general, the algorithm produced fewer positive classifications than the human reference and therefore operated with a low false-positive bias. Specifically, the automated positive ratio was slightly lower than the manual reference (median absolute difference = 0.42 percentage points). These results indicate that the epidermis-constrained quantification provides a reliable measurement of epidermal proliferation.

**Table 2:** Validation Results of the Manual or Auto Positive Ratio for 3 Patients and their Absolute Mean of the Relevant Values for Subtracting Manual from Automatic Counts (Auto-Manual), Relative Delta Δ_rel,i_, and Accuracy

Validation Case	Manual Positive Ratio	Auto Positive Ratio	Auto Manual	Δ_rel,i_	Accuracy
Ki-67 - 1	14.05%	13.41%	−0.64 pp	−4.6%	95.4%
Ki-67 - 2	8.26%	7.84%	−0.42 pp	−5.1%	94.9%
Ki-67 - 3	9.35%	8.98%	−0.37 pp	−4.0%	96.0%
Absolute mean			0.47 pp	4.57%	95.43%

Abbreviation: pp, percentage point.

#### Day-to-day variability

Furthermore, we assessed the time point–dependent changes in the outputs of the AI-based epidermis-constrained quantification by staining and analyzing the same samples at 2 different time points. In doing so, we observed no major discrepancies between the respective testing days (Table 3).

**Table 3:** Day-to-Day Variability of IHC Staining

Sample	1^st^ IHC Batch			2^nd^ IHC Batch			2^nd^ − 1^st^ Batch
	Positive Cells	Negative Cells	Ratio	Positive Cells	Negative Cells	Ratio	
1	47	1455	3,13%	47	1454	3,13%	0 pp
2	147	3799	3,73%	147	3712	3,81%	0.08 pp
3	171	2132	7,43%	169	2079	7,52%	0.08 pp
4	177	5164	3,31%	119	4264	2,72%	0.59 pp
Mean							0.19 pp

Same samples were stained and quantified at different time points.

Abbreviations: IHC, immunohistochemical; pp, percentage point.

However, because there were some changes between staining batches, we would like to acknowledge the limitations of this assessment, because deviations could also arise from changing sample properties. In paraffin sections, each slice represents a slightly different anatomical plane, making it difficult to distinguish whether potential differences reflect staining-day variability, algorithm performance, or natural tissue heterogeneity.

#### Control-slide verification

To further stress test the finalized workflow and to test whether the models would spuriously assign positive labels in the absence of true positives, we performed analyses on 10 negative control WSIs from 5 different patients, reflecting the 2 antigen-retrieval conditions used in this study. Across 32,711 cells, the combined AI models assigned the label “positive” to 29 cells in total, corresponding to a slide-level mean of 0.08% ± 0.06 SD, with no meaningful differences between retrieval buffers (n = 5 per condition; Tris/EDTA: 0.09% ± 0.04 SD; Citrate: 0.07% ± 0.07 SD). These results indicated negligible spurious positivity and further confirmed a low false-positive bias.

### Application to apoptosis quantification

**Figure 5:**
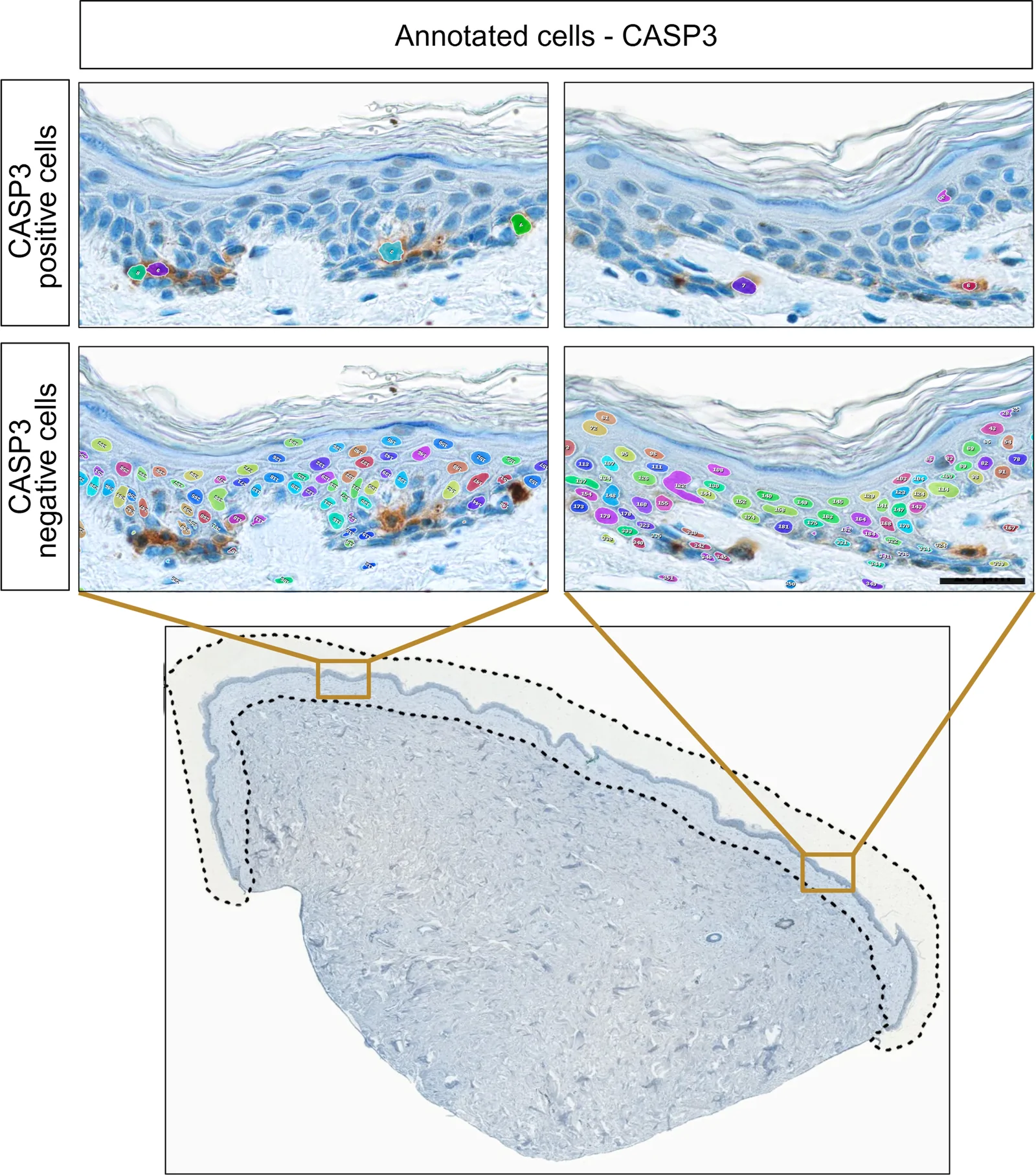
**CASP3-positive and -negative cells annotated by the AI model for cell detection (multicolored ellipses).** Apoptotic epidermal cells were annotated and classified by the AI model. In addition, cells in the dermal layer were annotated by the model and removed afterward using the epidermis-constrained quantification workflow. Bar = 25 μm. AI, artificial intelligence; CASP3, Caspase-3.

To complement our analysis of proliferation with an assessment of apoptosis in the epidermis, we applied our epidermis-constrained quantification model to samples stained for cleaved CASP3. This marker, similarly to Ki-67, produced a well-defined nuclear 3,3′-diaminobenzidine (DAB) signal in epidermal keratinocytes. The shared nuclear morphology allowed the AI model trained on Ki-67 to be reused for cell detection without additional training. Qualitative overlays confirmed accurate recognition of cleaved CASP3–positive and –negative nuclei within the epidermis (Figure 5).

Consistent with this rationale, the validation of cleaved CASP3 demonstrated close agreement between automated outputs and reference counts. Specifically, automated and manual positive ratios were nearly identical (median absolute difference = 0.04 percentage points) (Table 4) and demonstrated a feasible dual-marker assessment of proliferation and apoptosis in human skin.

**Table 4:** Validation Results for 3 Patients and their Absolute Mean of the Relevant Values for Auto-Manual Difference, Relative Delta, and Accuracy

Validation Case	Manual Positive Ratio	Auto Positive Ratio	Auto - Manual	Relative Delta	Accuracy
CASP3 - 1	2.50%	2.62%	+0.12 pp	+4.5%	95,5%
CASP3 - 2	1.92%	1.88%	−0.04 pp	−2.1%	97.9%
CASP3 - 3	1.78%	1.74%	−0.04 pp	−2.4%	97.6%
Absolute mean			0.07 pp	3.0%	97.0%

Abbreviations: CASP3, Caspase-3; pp, percentage point.

### HT reduces proliferation in epidermal cells

**Figure 6:**
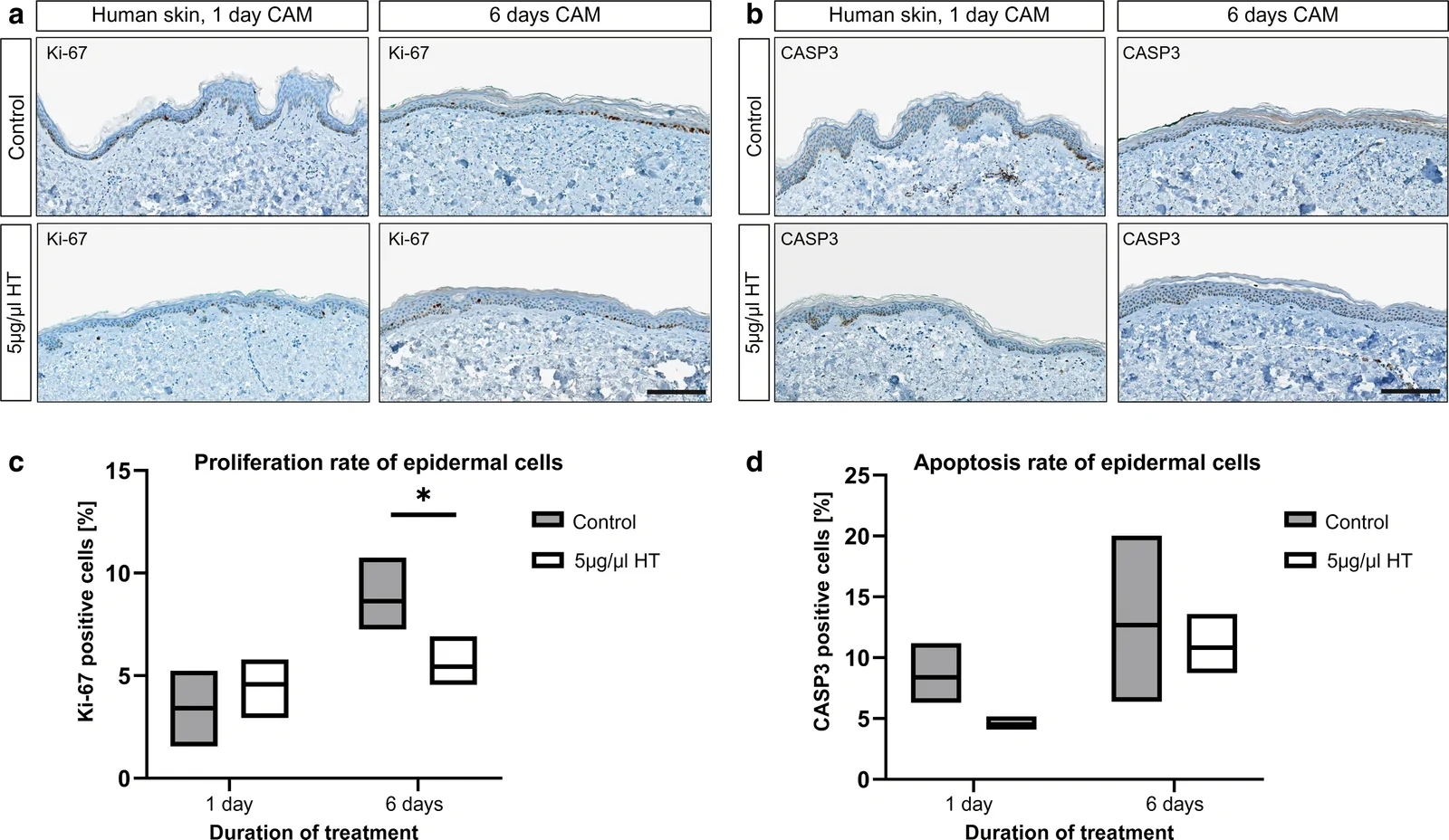
**Proliferation and apoptosis rates of epidermal cells after HT treatment (n = 3).** Data are shown after 1 day (denoted as 1d) and 6 days (denoted as 6d) of treatment. (**a**) Ki-67–labeled, proliferating cells, and (**b**) CASP3-labeled, apoptotic cells, after treatment with HT compared with the control group. (**c**) Quantification of the proportion of Ki-67–positive cells in the epidermis showed significantly reduced proliferation rates after 6 days of treatment with HT (*t*-test, ∗*P* = .016). (**d**) Quantification of the relative number of CASP3-positive cells in the epidermis showed no significant changes after treatment over time. Box plots represent the mean value with interquartile range. Bar = 200 μm. CASP3, Caspase-3; HT, hydroxytyrosol.

After successful validation and implementation of the AI-based epidermis-quantification workflow, we next evaluated its performance in a practical study setting. To this end, we used the CAM model as an established assay in our workgroup and investigated the effects of HT treatment on the regenerative capacity of human skin tissue (Figure 6).

A total of 12 samples derived from 3 patients were evaluated after 1 and 6 days of treatment with HT using the AI-based epidermis-constrained quantification. Representative HT-treated samples stained for Ki-67 as well as the respective control tissue are shown in Figure 6a. The quantification revealed a significant reduction (*P* < .05) in the proportion of proliferating epidermal cells after 6 days of treatment (Figure 6c). Conversely, skin samples stained for cleaved CASP3 (Figure 6b) displayed no significant changes in the apoptosis rates after HT treatment (Figure 6d).

## Discussion

In this study, we established and deployed an AI-based workflow for the quantitative analysis of IHC-stained human skin in a realistic experimental setting using a 3-dimensional in vivo model. By integrating an AI model for cell detection with a second AI model for epidermis recognition, we achieved objective, scalable, and reproducible quantification of epidermal proliferation and apoptosis. As an example application, we used this epidermis-constrained AI workflow to assess the impact of HT treatment on human skin.

This approach allowed us to address central challenges in histological image analysis, with a particular focus on the need for objective, high-throughput, and reproducible quantification of large datasets (Bankhead, 2022). A central goal was to test the trained models under practical study constraints rather than only in curated benchmarks. Our experimental setup provided biologically meaningful variation in terms of different patients, different analysis time points, and realistic staining variation, permitting evaluation at whole-slide scale. Within this environment, the combination of 2 AI models resulting in epidermis-constrained quantification minimized confounding by dermal cells, reduced false positives, and improved the interpretability of sample-level indices.

To evaluate the reliability of our workflow, we additionally analyzed slides lacking specific immunostaining but containing challenging endogenous background features such as melanin pigment, a well-known confounder that can mimic DAB-positive nuclei in brightfield imaging (Aung et al, 2015). Representative overlays and whole-slide analyses of negative control slides showed that our AI models did not misclassify background features such as melanin deposits or generate artificial signals, underscoring robustness against visually similar yet nonspecific signals. This is particularly relevant in dermatopathology, where endogenous pigments and background precipitates can challenge algorithmic specificity.

Further evidence of robustness is provided by the biologically and technically relevant staining variability inherent to our dataset, including differences in hematoxylin counterstaining and natural batch-to-batch and cell-to-cell heterogeneity across independently processed slides. Because these factors influence chromogen contrast, nuclear clarity, and background intensity, the maintained concordance between automated and manual counts across diverse samples indicated stable model performance under real-world IHC conditions.

A practical advantage of our workflow is its favorable balance between annotation effort and achieved performance. Despite relying on a relatively modest number of annotations—that is, approximately 5800 cells for the cell-detection model and fewer than 50 annotations for epidermis recognition—the models reached satisfying values for accuracy, precision, and specificity of ∼ 95% or higher. This high learning efficiency substantially reduces the time and annotation effort typically associated with generating training data for histopathology AI models (Bahadir et al, 2024).

Furthermore, the cell-detection model trained on Ki-67 was directly transferable to another nuclear marker with a similar DAB staining morphology (cleaved CASP3) without retraining, achieving even higher validation accuracy (∼97%, ∼1.5% above Ki-67). This underscores the potential for marker generalization when staining patterns are comparable. Although this strategy allowed us to avoid developing one model per antibody in this study, we note that retraining will be necessary when targeting antibodies with substantially different staining morphologies. Nevertheless, even in such cases, our 2-model design remains training efficient because only the cell-detection component requires retraining as long as the epidermis remains the structure of interest.

Our work represents a valuable contribution to extending the use of AI in dermatology beyond the field of oncology. Most existing AI-based applications in dermatopathology have primarily focused on standard H&E-stained slides (Ma et al, 2025). In contrast, our approach provides a reproducible method for analyzing immunohistochemistry, enabling the assessment of specific markers while simultaneously detecting information on general tissue morphology, such as epidermis recognition. A PubMed search conducted in March 2026 illustrates this gap: using the terms “artificial intelligence AND Ki-67 AND skin” yielded only 3 publications, and “artificial intelligence AND CASP3 AND skin” returned only 4. This scarcity highlights the relevance of our IHC approach.

Other existing studies that address AI-assisted IHC analysis in the skin differ substantially in scope and methodology. For example, Ding et al (2023) developed a deep-learning pipeline within QuPath to analyze skin immunohistochemistry, but their work primarily focused on software training procedures and required a seemingly more extensive manual setup. Our method, by contrast, offers a streamlined and scalable pipeline that reduces user-dependent variability and minimizes manual preprocessing. It generates standardized outputs (Excel, JSON, CSV) with object-level metrics and cell counts, complemented by automated visualization through JPEG exports and image overlays, enabling simultaneous extraction of marker-specific data and tissue-level morphological information.

Applying our workflow to HT-treated samples, a significant reduction in epidermal proliferation after 6 days of treatment, without a concomitant increase in apoptosis, was observed. Because the antiproliferative effect did not correlate with increasing rates of programmed cell death, this finding suggests a direct effect on cell cycle dynamics or alternative pathways. In addition, our findings are consistent with publications describing growth-suppressive or cell-cycle–modulating activities of HT in epithelial and melanoma systems (Aydar et al, 2017; Chen et al, 2023; Costantini et al, 2020; Granados-Principal et al, 2011). However, there are also studies reporting a positive effect of HT on proliferation (Kamil et al, 2020; Zrelli et al, 2011). This suggests context-dependent effects, reflecting differences in model systems, inflammatory status, and dosing. Therefore, further investigation for translational applications in wound repair is required. Together with our reliable analysis approach, our study adds value to existing, partly contradictory, literature on the effects of HT and demonstrates practical utility and feasibility of AI-based analyses in a treatment-response setting.

Overall, we found that the AI-based applications integrated into the IKOSA platform provide accurate and reproducible quantification of IHC-stained human skin with modest annotation effort. The output formats are user friendly and self-explanatory, facilitating straightforward downstream analysis and enabling integration into further applications. Given the method’s transferability and robustness across biological and technical variability, integration into established clinical workflows as a complementary diagnostic procedure appears feasible. The ability to capture biologically coherent treatment effects suggests that the epidermis-constrained AI workflow may be useful and extendable for research and translational work in dermatopathology.

## Materials and Methods

### Experimental setup

Human skin removed as part of plastic surgeries from female patients was used in this study. The patients provided written, informed consent, and the experiments were approved by the associated Ethics Committee. The tissue was processed by removing the subcutaneous fat and washing it in PBS before and after disinfection with 70% ethanol. Equally sized samples (Ø 8 mm) with a dermal and epidermal side were wounded and marked with green color.

The CAM assay was performed according to previous publications (Bichlmayer et al, 2022; Ettner-Sitter et al, 2024; Kauffmann et al, 2018). On day 7 or 8 of chick embryonic development, human skin biopsy punches were engrafted onto the CAM with the epidermis facing upward.

To test the effects of HT on the proliferation and apoptosis of epidermal cells, 5 μg/μl HT dissolved in 10% glycerol in pure H_2_O was applied topically and distributed across the skin samples daily, with the final application occurring 3 hours prior to excision. The control group was treated with a 10% glycerol solution. For these experiments, 12 samples from 3 patients were evaluated using the AI models described below. These samples were not part of the AI model training or validation set.

### (Immuno)histology

The tissue samples were excised after 1 and 6 days of cultivation on the CAM and fixed in 4% paraformaldehyde in PBS, pH 7.4, for approximately 24 hours at 4 °C, followed by storage in 0.02% sodium azide until dehydration. Dehydration was performed using an ascending isopropanol series, 2 incubations in xylene, and subsequent incubation in paraffin in the TP10120 automated tissue processor (Leica Biosystems, Nußloch, Germany), and the samples were embedded in paraffin using the Leica EG1150 H paraffin embedding station (Leica Biosystems). For immunohistochemistry, 6-μm sections were cut with the Leica RM2245 semimotorized rotary microtome (Leica Biosystems).

Proliferating cells within the epidermis were visualized by labeling the proliferation marker Ki-67 and cleaved CASP3 to identify epidermal cells undergoing apoptosis.

IHC staining was performed after deparaffinization and rehydration of the tissue slides using the ZytoChem Plus (HRP) Broad Spectrum DAB Kit (Zytomed Systems GmbH, Berlin, Germany). After heat-mediated antigen retrieval, inactivation of endogenous peroxidases with 3% hydrogen peroxide in 70% ethanol, and subsequent blocking with the kit’s blocking solution, the primary antibodies were incubated overnight at 4 °C diluted in 1% BSA in PBS (Table 5). The secondary antibody, streptavidin-complex, and DAB from the kit were applied on the next day. PBS washing was performed between the incubation steps. Slides were counterstained with hematoxylin Gill no.3 (Sigma-Aldrich, Saint Louis, MO), blued in warm running tap water, and mounted using AquaTex (Merck KGaA, Darmstadt, Germany). For each staining procedure, a negative control was included by incubating sections with 1% BSA solution without the primary antibody overnight. The remaining IHC protocol remained unchanged, allowing assessment of staining quality and ensuring that no background signal resulted from nonspecific interactions. For the respective patients, separate staining procedures were performed.

**Table 5:** Used Primary Antibodies, their Vendor, and Respective Dilutions and Antigen Retrieval

Marker	Host/Isotype	Clonality	Vendor/Catalog Number	Dilution	Antigen Retrieval
Ki-67 (SP6)	Rabbit IgG	Monoclonal	Abcam, ab16667	1:200	Tris/EDTA (pH 9)
CASP3 (cleaved) (Asp175)	Rabbit	Polyclonal	Cell Signaling Technology, #9661	1:100	Citrate (pH 6)

Abbreviation: CASP3, Caspase-3.

### Image acquisition

Slides were digitized using a PreciPoint M8 Slide Scanner with a ×40 objective (PreciPoint GmbH, Munich, Germany) and stored as WSI.

### Image analysis pipeline

The IKOSA image analysis platform allows the creation of image segmentation models using state-of-the-art CNN architectures without coding. Such models can be trained and applied to detect objects, such as cell nuclei, or demarcate specific areas, such as tumors, necrosis, or skin layers. Trained AI models can then be deployed to analyze an entire WSI or a specific ROI.

The proposed image analysis pipeline consists of 3 stages: (i) epidermis detection, (ii) cell detection and classification of Ki-67– and cleaved CASP3–positive nuclei, and (iii) fusion of outputs to quantify epidermal cell ratios at the sample level. Data processing was assisted by Microsoft Excel and Python (version 3.14, custom scripts available upon request). Despite a comparatively limited training set and modest annotation effort, the models yielded strong validation performance (see below).

### AI cell detection: training and annotation

To recognize, annotate, and classify cells in our WSIs, we trained an AI model for cell detection using tissue slides stained for Ki-67 from 2 sources: human skin and pancreatic carcinoma tissue. Pancreatic tissue served as a source for pretraining, providing a broader range of nuclear DAB morphologies and improved positive class representation. We then incorporated skin WSIs into the training to adapt the AI model for application in human skin. The final training dataset (Table 6) consisted of 5 human skin samples from different patients and 4 tissue slides of human pancreatic carcinoma tissue. Importantly, all analyses and biological inferences are based on skin slides only.

**Table 6:** Training Set Used to Train the AI Model for Cell Detection

Sample	Number of ROIs	Annotated Positive Cells	Annotated Negative Cells	Total Annotations
Skin-1	1	4	232	236
Skin-2	5	49	919	968
Skin-3	6	12	322	334
Skin-4	9	18	102	120
Skin-5	13	86	399	485
Pancreatic tissue	21	748	2876	3624
Total	55	917	4850	5767

Abbreviations: AI, artificial intelligence; ROI, region of interest.

ROIs were manually placed as randomly as possible across different tissue regions to ensure that the model encountered a broad range of histological features, including the epidermis as the structure of interest but also dermis, skin appendages such as hair follicles, and slide background. For the pancreatic carcinoma slides, ROIs were likewise assigned manually and were typically placed in more cell-dense areas. Within these ROIs, cell nuclei were manually annotated and classified as Ki-67 positive or negative. In the skin samples, only epidermal nuclei were annotated, because dermal nuclei were not relevant to the intended biological endpoint. Isotype control slides were not used for algorithm training to avoid artificially inflating negative-class pixels or pushing the model to overfit to background variations instead of learning real signal morphology.

### AI cell detection: training validation

For training validation, a total of 16 ROIs distributed across 7 different images were used. These validation ROIs were not part of the training dataset but had been manually annotated beforehand to enable internal performance assessment. In total, 3211 annotated cells were available for this validation step, allowing comparison between automated predictions and ground-truth manual labels. Results of the training validations are shown in the Results section.

### AI epidermis recognition: training and annotation

To restrict subsequent quantitative analyses to the epidermis as our structure of interest, we trained a dedicated semantic-segmentation model for epidermis detection. The training dataset consisted of 5 WSIs of IHC-stained human skin. ROIs were again placed randomly to capture different tissue contexts, including epidermis, dermis, adnexal structures, and background. In these 43 ROIs, the epidermis (excluding the stratum corneum) was manually annotated with maximal accuracy. In some ROIs, the epidermis was subdivided into multiple annotations to maintain delineation accuracy (Table 7). The training was performed using a downscale factor of 3 as a training parameter for semantic segmentation.

**Table 7:** Training Set Used to Train the AI Model for Epidermis Recognition

Sample	Number of ROIs	Number of Epidermis Annotations
Skin-1	5	5
Skin-2	6	8
Skin-3	10	12
Skin-4	10	13
Skin-5	12	9
Total	43	47

Abbreviations: AI, artificial intelligence; ROI, region of interest.

### AI epidermis recognition: validation

The validation integrated into the training was performed on 16 manually assigned ROIs placed at different positions across 2 independent samples. This validation set was not included in the training dataset. Instead, the epidermis within each ROI was manually annotated beforehand to serve as a ground-truth reference, allowing direct comparison between the AI-generated segmentation and the corresponding manual annotations, as described earlier.

### Epidermis-constrained quantification: combined workflow

Because the output of the epidermis-recognition model included precise positional coordinates of the epidermis within each IHC-stained sample, it could ideally be combined with the AI cell-detection model to restrict cell detection and counting to the epidermal compartment. This combined workflow produced per-sample positive ratios for Ki-67 and cleaved CASP3 (number of positive cells divided by the total number of detected cells within the epidermis). Although the model for cell detection did not initially “know” to count epidermal cells exclusively, it integrated with the semantic segmentation model, achieving epidermis-constrained quantification without retraining.

### Epidermis-constrained quantification: validation

For validation of the combined workflow, we compared automated readouts against manual counts on representative validation slides from 3 patients. For each patient, at least 3 ROIs were counted manually, and the same ROIs were analyzed using the combined workflow for epidermis-constrained quantification. Agreement was evaluated at the level of the positive cell ratio (positive cells/total) because positive and negative labels were learned independently by the AI cell-detection model.

For each case, we calculated the following:

• Mean accuracy as 100% − mean(Δ_rel,i_);
• Mean difference as $\frac{1}{n}\sum_{i=1}^{n}\left(Ai-Mi\right)$ across all cases, representing the systematic over or underestimation of the manual ratio by the automated method, in percentage points;
• Median absolute difference as median absolute difference = median_i_ (|*Ai – Mi*|) across cases and describes the typical magnitude of the discrepancy between automated and manual indices, irrespective of the direction; and
• Relative delta as Δ_rel,i_ = $\frac{Ai-Mi\times 100\%}{Mi}$, where *Ai* is the automated and *Mi* the manual positive ratio.

## Ethics Statement

All patients provided written and informed consent, and the study was approved by the Ethics Committee of the University of Regensburg (ethics approval number: 22-3199-101). We used a 3R-compliant model, reducing the need for animal testing.

## Data Availability Statement

The datasets generated and/or analyzed during this study are available from the corresponding author, VR, upon request. Additional information required to reproduce the findings reported in this study is also available from the corresponding author upon request.

## ORCIDs

Valeria Mei: http://orcid.org/0009-0004-6483-3244

Sandra Maurer: http://orcid.org/0009-0002-8928-5657

Philipp Kainz: http://orcid.org/0000-0001-7970-370X

Andreas Ettner-Sitter: http://orcid.org/0009-0008-6443-2597

Thea Bauer: http://orcid.org/0009-0009-2144-6283

Thiha Aung: http://orcid.org/0009-0005-0591-3567

Silke Haerteis: http://orcid.org/0000-0002-9440-3307

Vera Rötzer: http://orcid.org/0009-0006-6370-121X

## Conflict of Interest

PK is managing director and shareholder of KOLAIDO GmbH. The remaining authors state no conflict of interest.
